# Otoprotective Effect of 2,3,4′,5-Tetrahydroxystilbene-2-*O*-β-d-Glucoside on Gentamicin-Induced Apoptosis in Mouse Cochlear UB/OC-2 Cells

**DOI:** 10.3390/molecules25133070

**Published:** 2020-07-06

**Authors:** Yu-Hsuan Wen, Jia-Ni Lin, Rong-Shuan Wu, Szu-Hui Yu, Chuan-Jen Hsu, Guo-Fang Tseng, Hung-Pin Wu

**Affiliations:** 1 Institute of Medical Sciences, Tzu Chi University, Hualien 970374, Taiwan; hatsuyuki2001@yahoo.com.tw (Y.-H.W.); guofang@mail.tcu.edu.tw (G.-F.T.); 2 Department of Otolaryngology, Head and Neck Surgery, Hualien Tzu Chi Hospital, Buddhist Tzu Chi Medical Foundation, Hualien 970473, Taiwan; 3 Department of Otolaryngology, Head and Neck Surgery, Taichung Tzu Chi Hospital, Buddhist Tzu Chi Medical Foundation, Taichung 427213, Taiwan; neonlin0939@gmail.com (J.-N.L.); cjhsu@ntu.edu.tw (C.-J.H.); 4 The Affiliated Senior High School of National Chung Hsing University, Taichung 412011, Taiwan; clairewu1022@gmail.com; 5 Department of Music, Tainan University of Technology, Tainan 710302, Taiwan; t50311@gm.tut.edu.tw; 6 Department of Otolaryngology, National Taiwan University Hospital, Taipei 100225, Taiwan; 7 Department of Anatomy, Tzu Chi University, Hualien 970374, Taiwan; 8 School of Medicine, Tzu Chi University, Hualien 970374, Taiwan

**Keywords:** otoprotection, reactive oxygen species (ROS), 2,3,4′,5-tetrahydroxystilbene-2-*O*-β-d-glucoside (THSG), *Polygonum multiflorum* Thunb., gentamicin, UB/OC-2 cells

## Abstract

Excessive levels of reactive oxygen species (ROS) lead to mitochondrial damage and apoptotic cell death in gentamicin-induced ototoxicity. 2,3,4’,5-Tetrahydroxystilbene-2-*O*-β-d-glucoside (THSG), a bioactive constituent, isolated from *Polygonum multiflorum* Thunb., exhibits numerous biological benefits in treating aging-related diseases by suppressing oxidative damage. However, its protective effect on gentamicin-induced ototoxicity remains unexplored. Therefore, here, we aimed to investigate the otoprotective effect of THSG on gentamicin-induced apoptosis in mouse cochlear UB/OC-2 cells. We evaluated the effect of gentamicin and THSG on the ROS level, superoxide dismutase (SOD) activity, mitochondrial membrane potential, nuclear condensation, and lactate dehydrogenase (LDH) release, and the expression of apoptosis-related proteins was assessed to understand the molecular mechanisms underlying its preventive effects. The findings demonstrated that gentamicin increased ROS generation, LDH release, and promoted apoptotic cell death in UB/OC-2 cells. However, THSG treatment reversed these effects by suppressing ROS production and downregulating the mitochondrial-dependent apoptotic pathway. Additionally, it increased the SOD activity, decreased the expression of apoptosis-related proteins, alleviated the levels of the apoptotic cells, and impaired cytotoxicity. To the best of our knowledge, this is the first study to demonstrate that THSG could be a potential therapeutic option to attenuate gentamicin-induced ototoxicity.

## 1. Introduction

The World Health Organization has estimated that there are about 466 million people (approximately 6.1% of the world’s population) with disabling hearing loss [1]. Hearing loss can occur due to aging, noise exposure, and ototoxic drugs (e.g., aminoglycosides, platinum-based chemotherapeutic agents, loop diuretics, nonsteroidal anti-inflammatory drugs) that cause overproduction of reactive oxygen species (ROS) and/or reduction of cochlear blood flow [2,3,4]. Cochleae are vulnerable to oxidative stress on account of the high metabolic demands of hair cells in reaction to stimulation [5]. Moreover, ROS contribute to cellular dysfunction, including DNA damage and lipid peroxidation, leading to cochlear degeneration [6].

Gentamicin, an aminoglycoside antibiotic, is commonly used to treat infections caused by aerobic Gram-negative and some Gram-positive bacteria [7]. However, it has been reported to exert ototoxic side effects leading to cochlear and/or vestibular damage [8]. Mechanistically, the ototoxic effects of gentamicin are mediated by the oxidative stress pathway [9,10], apoptosis [11,12,13], autophagy [14,15], and the Akt survival pathway [16,17,18]. Apoptosis plays an important role in maintaining intracellular homeostasis and participates in the pathogenesis of hearing loss [19]. Excessive ROS production by ototoxic drugs is associated with hair cell damage via the apoptotic pathway [20]. In addition, it has been shown that antioxidants attenuated gentamicin-induced hair cell damage, suggesting a possible relationship between ROS and gentamicin-induced ototoxicity [21].

*Polygonum multiflorum* Thunb., also known as “He-Shou-Wu” in the East and “Fo-ti” in the West, is commonly used in traditional Chinese medicine for its antiaging effects [22,23,24]. It is also used in medicinal food to improve health. The stilbene glucoside 2,3,4′,5-tetrahydroxystilbene-2-*O*-β-d-glucoside (THSG) is a bioactive component extracted from the root of *P. multiflorum* Thunb. Several pharmacological studies have demonstrated that THSG exhibits antioxidative capacity [25], attenuates inflammation, including reduction of the levels of inflammatory factors in atherosclerotic rat serum and lysophosphatidylcholine (LPC)-mediated induction of inflammatory factors in microglia [26,27], and eliminates the apoptotic effects in ischemia/reperfusion injury and LPC-induced injury [28,29]. In addition, it has also been shown that THSG improves blood flow and ameliorates vascular senescence in spontaneously hypertensive rats [30].

Moreover, recent studies have reported the protective effect of THSG on oxidative stress-induced cellular damage. However, the relationship between gentamicin ototoxicity and THSG remains unknown. In this study, we investigated the otoprotective effect of THSG in the gentamicin-treated mouse cochlear cell line and elucidated the molecular mechanisms underlying the protective effects of THSG. The findings demonstrated the potential of THSG to treat gentamicin-induced ototoxicity.

## 2. Results

### 2.1. Gentamicin Suppresses Cell Viability and Induces Cell Cytotoxicity in University of Bristol/Organ of Corti-2 (UB/OC-2) Cells

**Figure 1:**
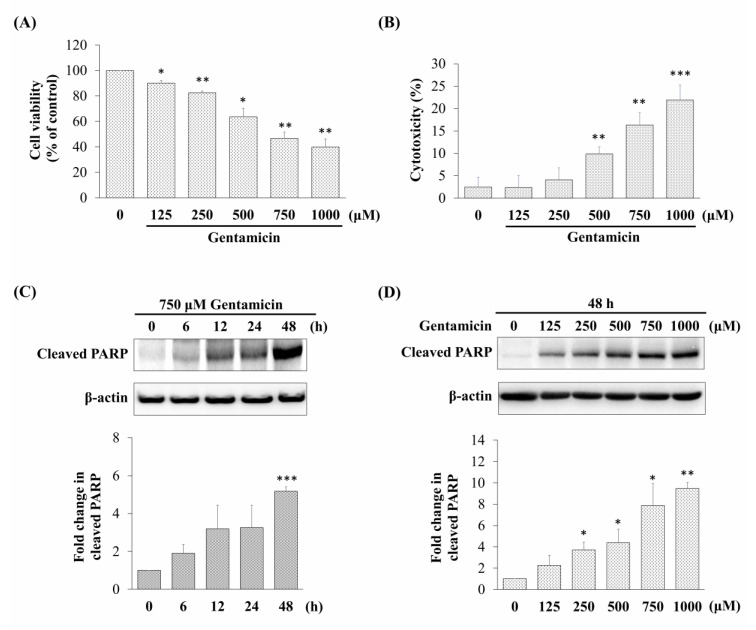
Effect of gentamicin on cell viability, cytotoxicity, and cleaved poly (ADP-ribose) polymerase (PARP) expression in mouse cochlear UB/OC-2 cells. The damage to the cells at various gentamicin concentrations (48 h) was assessed by (**A**) 3-(4,5-dimethylthiazol-2-yl)-2,5-diphenyltetrazolium bromide (MTT) assay and (**B**) lactate dehydrogenase (LDH) release assay. Relative expression levels of cleaved PARP detected by western blotting in cells treated with (**C**) 750 μM gentamicin at different exposure times and (**D**) different concentrations of gentamicin for 48 h. All data are expressed as the mean ± standard deviation from three independent experiments. * *p* < 0.05, ** *p* < 0.01, *** *p* < 0.001 vs. the control group.

The assessment of cell viability showed that gentamicin inhibited the cell viability in a dose-dependent manner. The viability of cells treated with 750 μM gentamicin was reduced to 46.64 ± 4.90% in comparison with the control cells (not treated with gentamicin) (Figure 1A). In addition, we measured the cellular cytotoxicity by measuring the lactate dehydrogenase (LDH) activity after 48 h of gentamicin exposure in various concentrations (125–1000 μM). As shown in Figure 1B, gentamicin significantly increased the release of LDH in a dose-dependent manner. These results showed that gentamicin could suppress the cell viability and induce cytotoxicity in mouse cochlear UB/OC-2 cells.

### 2.2. Gentamicin Promotes Apoptotic Cell Death in UB/OC-2 Cells

Based on the cell viability in the MTT assay, we chose 750 μM of gentamicin that resulted in 50% viability inhibition as an ototoxicity inducer in the subsequent experiments. In order to elucidate the mechanisms involved in the toxic effect of gentamicin via activation of apoptosis, we detected the relative expression of cleaved poly (ADP-ribose) polymerase (PARP), an indicator of apoptosis. As shown in Figure 1C,D, the relative expression levels of cleaved PARP were significantly increased after gentamicin treatment in a time-dependent and dose-dependent manner. Taken together, these data suggested that gentamicin might induce UB/OC-2 cell apoptosis.

### 2.3. THSG Protects UB/OC-2 Cells against Gentamicin-Induced Oxidative Stress

**Figure 2:**
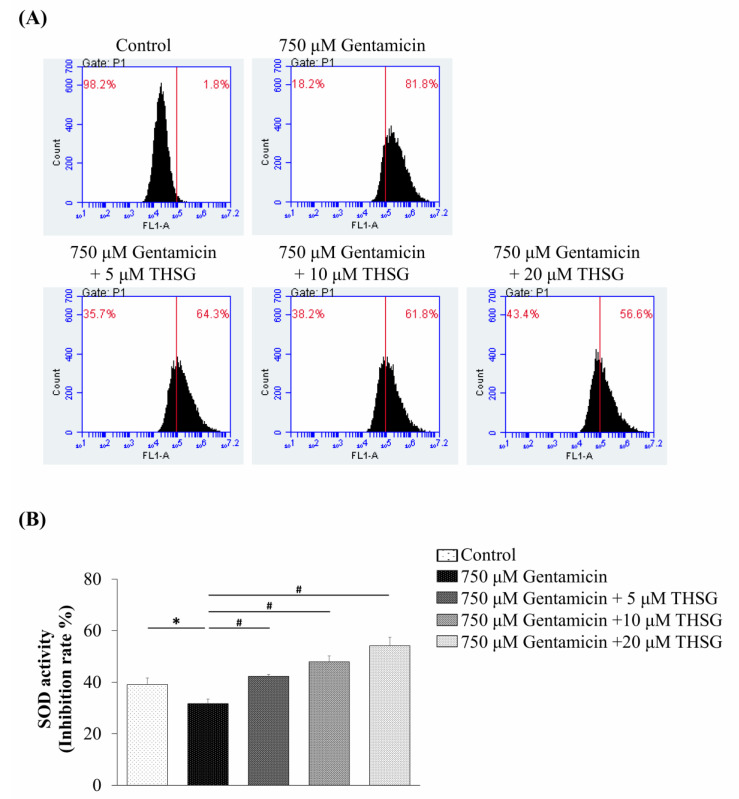
Effects of 2,3,4’,5-tetrahydroxystilbene-2-*O*-β-d-glucoside (THSG) on gentamicin-induced reactive oxygen species (ROS) generation and antioxidant enzyme activities in mouse cochlear UB/OC-2 cells. The cells were treated with various THSG concentrations followed by 750 μM gentamicin to assess (**A**) ROS levels by flow cytometry using 2′,7′-dichlorofluorescein diacetate (DCFDA) fluorescent dye and (**B**) the superoxide dismutase (SOD) activity. All data are expressed as means ± standard deviation from three independent experiments. * *p* < 0.05 vs. the control group; ^#^ *p* < 0.05 vs. the gentamicin-treated group.

In our previous report, no effect on the survival of UB/OC-2 cells treated with THSG at different concentrations (5–40 μM) for 48 h was seen [31]. To further investigate whether THSG protects gentamicin-treated UB/OC-2 cells from oxidative stress, the levels of ROS were measured by flow cytometry using the indicator dye 2′,7′-dichlorofluorescein diacetate (DCFDA). The fluorescence signal intensity of DCFDA staining was boosted in the gentamicin-treated group compared to the untreated group. The DCFDA staining showed that the THSG treatment decreased the fluorescence intensity markedly in gentamicin-treated cells (Figure 2A). Further, to confirm the effect of THSG on oxidative stress, we measured the antioxidative enzyme activity of superoxide dismutase (SOD). The results showed that the SOD activity in the three THSG-treated groups (treated with 5, 10, and 20 µM of THSG) was significantly higher (42.28 ± 0.72%, 47.90 ± 2.27%, and 54.16 ± 3.32%, respectively) compared to the gentamicin-treated group (31.67 ± 1.75%) (Figure 2B). These results demonstrated that THSG could suppress gentamicin-induced ROS production and increase SOD activity in UB/OC-2 cells.

### 2.4. THSG Impairs Gentamicin-Induced Mitochondrial-Dependent Apoptotic Pathway and Prevents Cell Death in UB/OC-2 Cells

**Figure 3:**
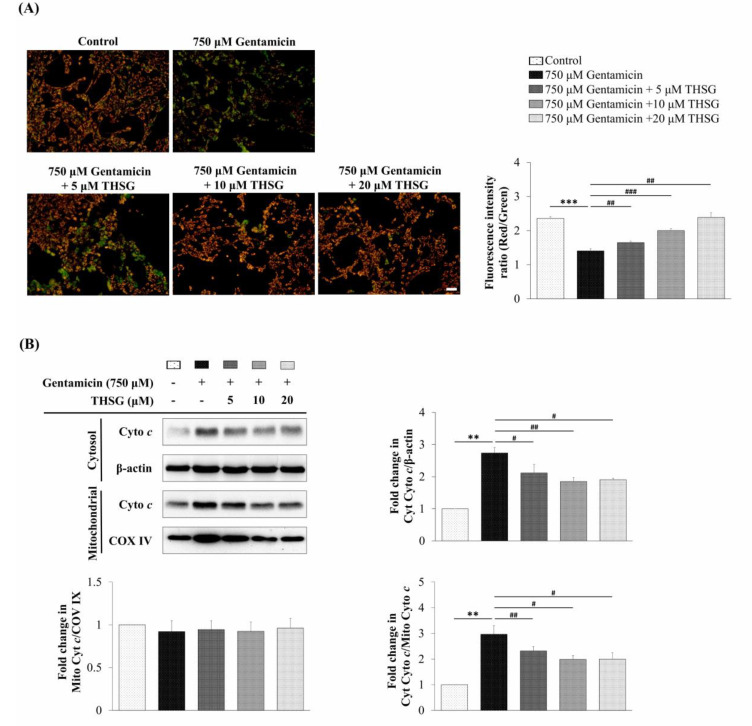
Effects of THSG on gentamicin-induced mitochondrial membrane potential and cytochrome *c* protein expression in mouse cochlear UB/OC-2 cells. The cells were treated with different concentrations of THSG for 6 h, followed by 750 μM gentamicin for 24 h. (**A**) Mitochondrial membrane potential was determined by the 5,5′,6,6′-tetrachloro-1,1′,3,3′-tetraethylbenzimidazolylcarbocyanine iodide (JC-1) fluorescence dye. Scale bar = 50 μm. Images were obtained under a fluorescence microscope. (**B**) Cytochrome *c* was analyzed by western blotting in cytosolic and mitochondrial fractions. β-actin and COX IV were loading controls for cytosolic and mitochondrial fractions, respectively. All data are expressed as the means ± standard deviation from three independent experiments. ** *p* < 0.01, *** *p* < 0.001 vs. the control group; ^#^ *p* < 0.05, ^##^ *p* < 0.01, ^###^ *p* < 0.001 vs. the gentamicin-treated group.

To monitor the changes of mitochondrial membrane potential, fluorescence images of UB/OC-2 cells with 5,5′,6,6′-tetrachloro-1,1′,3,3′-tetraethylbenzimidazolylcarbocyanine iodide (JC-1) dye were observed under a fluorescence microscope. JC-1 dye produces orange-red fluorescence in healthy mitochondria but green fluorescence at low membrane potential. As shown in Figure 3A, the gentamicin only group displayed a relatively low ratio of red to green fluorescence. The THSG in gentamicin-treated groups exhibited an increased ratio of red to green fluorescence compared to the gentamicin-alone group. The results showed that THSG recovered the gentamicin-induced loss of mitochondrial membrane potential. As cytochrome *c* is a key mediator of the apoptosis pathway, we next evaluated the gentamicin-stimulated cytochrome *c* release from mitochondria. It was observed that THSG treatment blocked the release of cytochrome *c* from mitochondria to the cytosol (Figure 3B). Collectively, these results showed that THSG evaded gentamicin-induced oxidation stress and downregulated mitochondria-dependent apoptosis in UB/OC-2 cells.

**Figure 4:**
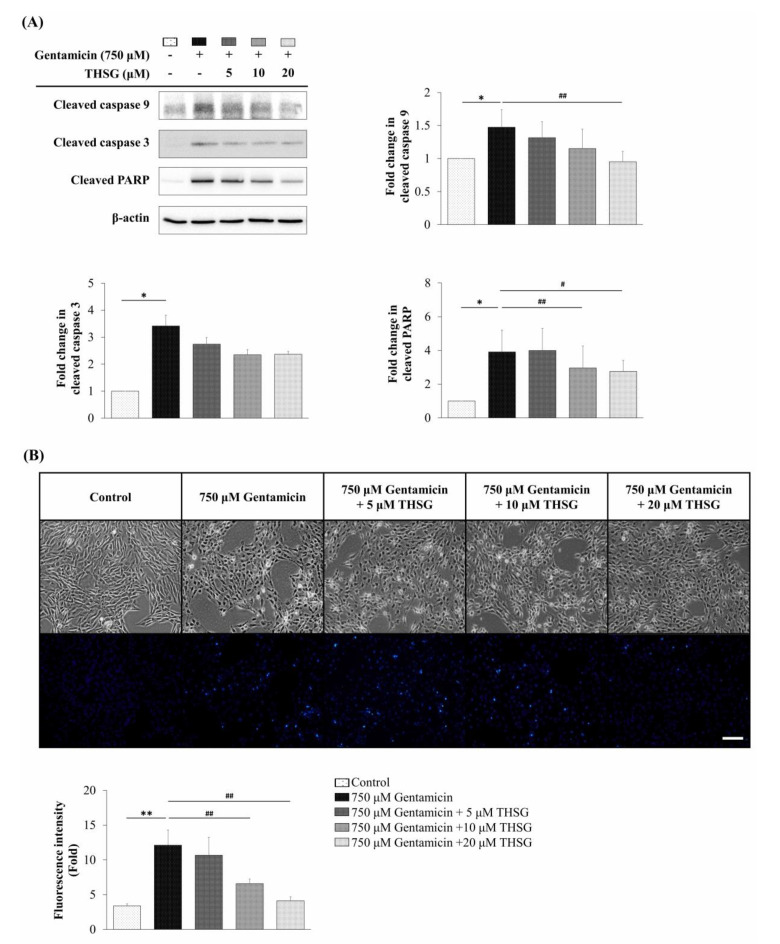
Effects of THSG on gentamicin-induced apoptotic protein expression and nuclear condense in mouse cochlear UB/OC-2 cells. Cells were treated with various THSG concentrations for 6 h, followed by 750 μM gentamicin for 48 h. (**A**) Protein levels of cleaved caspase 9, cleaved caspase 3, and cleaved PARP were analyzed by western blotting. (**B**) Nuclear apoptotic changes were assessed using Hoechst 33258 fluorescence dye. Scale bar = 50 μm. Images were obtained using a fluorescence microscope. All data are expressed as the means ± standard deviation from three independent experiments. * *p* < 0.05, ** *p* < 0.01 vs. the control group; ^#^ *p* < 0.05, ^##^ *p* < 0.01 vs. the gentamicin-treated group.

To confirm the apoptotic effects of THSG on mitochondrial-dependent apoptosis, the protein expression of cleaved caspase 9, cleaved caspase 3, and cleaved PARP were analyzed by western blotting. The results showed that THSG suppressed the cleaved levels of caspase 9, caspase 3, and PARP after gentamicin treatment (Figure 4A). Additionally, the nuclear staining using Hoechst 33258 dye showed a lower fluorescence intensity of Hoechst 33258 in the THSG-treated groups than in the gentamicin-alone group (Figure 4B) indicating reduced nuclear condensation.

**Figure 5:**
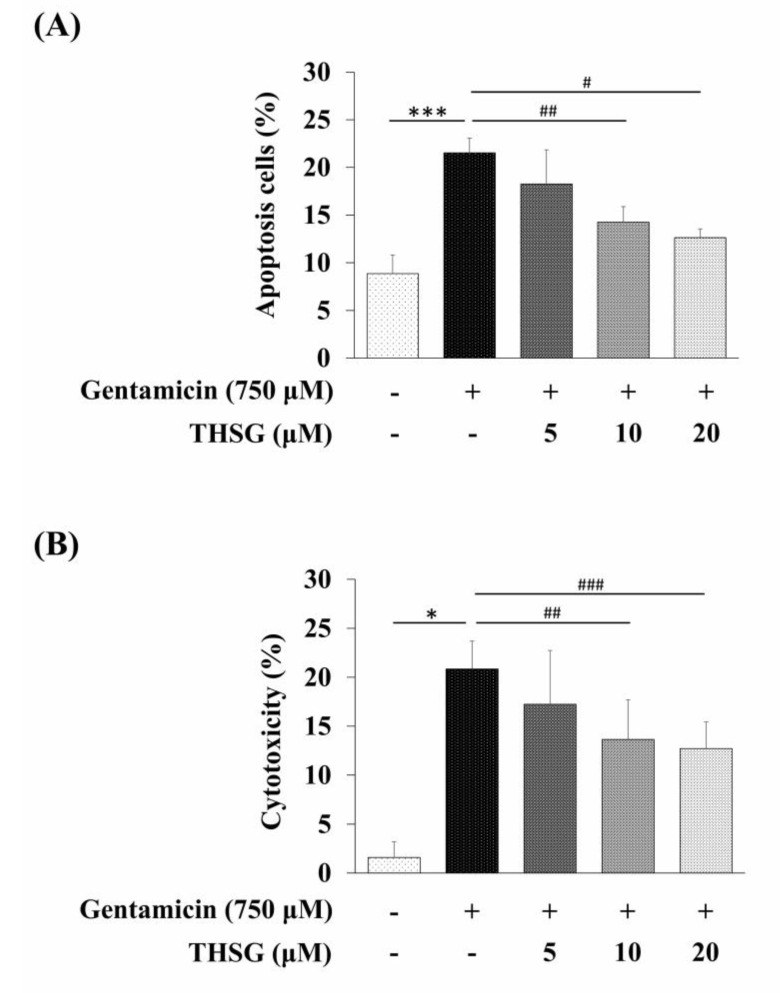
Effects of THSG on gentamicin-induced apoptosis and cytotoxicity in mouse cochlear UB/OC-2 cells. Cells were treated with various THSG concentrations for 6 h, followed by 750 μM gentamicin for 24 h. (**A**) The percentage of apoptotic cells was detected by Annexin V and propidium iodide (PI) staining. (**B**) Cytotoxicity was monitored by measuring LDH release. All data are expressed as the means ± standard deviation from three independent experiments. * *p* < 0.05, *** *p* < 0.001 vs. the control group; ^#^ *p* < 0.05, ^##^ *p* < 0.01, ^###^ *p* < 0.001 vs. the gentamicin-treated group.

We analyzed the population of apoptotic cells using Annexin V and propidium iodide (PI) double staining. The results revealed that the percentage of apoptosis was significantly lower in the THSG-treated groups as compared to the gentamicin-alone group (Figure 5A). To investigate the cytotoxic potential of THSG, the LDH cytotoxicity assay was used to check cell cytotoxicity. As shown in Figure 5B, the release of LDH was reduced in the THSG-treated groups compared to the gentamicin-alone group. Collectively, these results indicated that THSG treatment could decrease gentamicin-mediated apoptotic cell death via mitochondrial-dependent apoptosis in UB/OC-2 cells.

## 3. Discussion

To the best of our knowledge, this is the first study describing the protective effect of THSG against gentamicin-induced ototoxicity in mouse cochlear UB/OC-2 cells via inhibition of mitochondrial-dependent apoptosis signaling pathways. Our results indicated that gentamicin could decrease cell viability, increase cytotoxicity, stimulate ROS generation, reduce mitochondrial membrane potential, and induce apoptotic cell death in mouse cochlear UB/OC-2 cells. On the contrary, the administration of THSG could protect mouse cochlear UB/OC-2 cells from gentamicin-induced cell death by downregulating ROS production, upregulating SOD activity, suppressing mitochondrial depolarization, and inhibiting the mitochondrial-dependent apoptotic signaling pathway. These results suggested that THSG could attenuate the cochlear cell damage caused by gentamicin.

In biomedical research, cell lines are used as models for various purposes in vitro. There are few immortalized cell lines derived from the auditory sensory organ such as the House Ear Institute-Organ of Corti 1 (HEI-OC1), University of Bristol/Organ of Corti-1 (UB-OC-1) and UB/OC-2 cells. The cell line UB/OC-2, a conditionally immortalized mouse cochlear cell line derived from E13 embryos [32,33], has been used to study hereditary hearing loss [34], cell differentiation [35], ototoxicity [36] and otoprotection [31].

*P. multiflorum* Thunb. containing stilbene glucosides and anthraquinones as the major active constituents [37] has been widely used in Chinese herbal medicine for its health promoting and hair-blackening effects [38]. In 1975, Hata et al. [39] isolated THSG, which comprises a polyhydroxy stilbene, and a glycoside structure from the extract of *P. multiflorum* Thunb. Several studies have demonstrated the antioxidative capacity of THSG [25], and its antioxidant potential could be ascribed to its hydroxyl groups [40]. Lin et al. reported that THSG relieved adriamycin-induced focal segmental glomerulosclerosis through activation of the nuclear factor erythroid 2-related factor 2 (Nrf2)-Kelch-like ECH-associated protein 1 (Keap1) antioxidant pathway [41] and protected osteoblasts against H_2_O_2_-induced oxidative damage by regulating Nrf2 and nuclear factor kappa-light-chain-enhancer of activated B cells (NF-κB) signaling pathways [42]. It has also been shown that THSG inhibits glutamate-induced neurotoxicity via suppression of ROS production [43]. Our previous study reported that THSG has free radical scavengers and activates the Nrf2 signaling pathway to mitigate H_2_O_2_-induced ototoxicity [31]. Here, this study demonstrated that THSG suppressed gentamicin-induced ototoxicity by inhibiting ROS production and activating the antioxidant enzyme, SOD (Figure 2). However, future investigations are required to better clarify the antioxidant properties of THSG in oxidative stress-induced damage and the protective effects of THSG from drug-induced ototoxicity.

Aminoglycoside antibiotics are used widely for the treatment of bacterial infections because of their effectiveness and lower drug prices. However, the use of these agents is limited by their side effects, such as ototoxicity and nephrotoxicity [8]. Moreover, ROS formation has been shown to be related to aminoglycoside antibiotics-induced hair cell damage [44]. Therefore, co-administration of an aminoglycoside antibiotic and an antioxidant agent has been developed as a novel therapeutic approach for reducing aminoglycoside-induced ototoxicity at low cost without compromising the antimicrobial activity.

Apoptosis has been conceded to be an important part of aminoglycoside-induced ototoxicity [45]. Deregulation of apoptosis can contribute to protecting hair cells from ototoxicity [20]. Gentamicin is one of the most commonly used antibiotics that has been reported to cause vestibular dysfunction and cochlear damage, which may result in balance disorders, tinnitus, and hearing loss [4]. According to the previous study of El Mouedden et al., gentamicin treatment caused a higher ratio of apoptosis than other aminoglycosides under the same conditions [46]. In the present study, like several others [16,47,48], we found that gentamicin exposure in UB/OC-2 cells leads to apoptotic cell death (Figure 1 and Figure 5).

**Figure 6:**
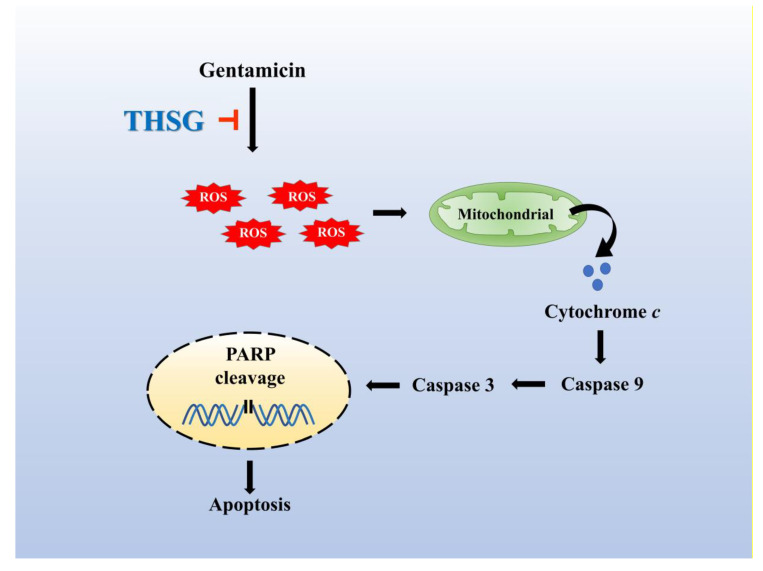
The otoprotective mechanism of THSG in alleviating gentamicin-induced UB/OC-2 cell apoptosis. THSG treatment impairs gentamicin-induced apoptosis by reducing ROS production, stabilizing mitochondrial membrane potential, and downregulating mitochondrial-dependent apoptotic protein expression.

Excess ROS levels may contribute to mitochondrial damage, including impairment of RNA translation, protein synthesis, and mitochondrial membrane permeability, and cause mitochondrial-dependent apoptosis [49,50,51]. We showed that ROS was significantly increased after 2 h of exposure to gentamicin, and THSG reduced the gentamicin-induced ROS generation (Figure 2A). The present study depicts early ROS production in gentamicin-induced toxicity that could have been triggered by lysosomal membrane permeabilization, followed by mitochondrial dysfunction [52]. Studies have shown that gentamicin causes apoptosis through the mitochondrial-dependent intrinsic apoptotic pathway but not the extrinsic apoptotic pathway [53,54,55]. Furthermore, cytochrome *c*, a caspase activator, is used to characterize the mitochondrial-dependent apoptotic pathway when released from mitochondria to cytosol, followed by the activation of caspase 9, caspase 3, and PARP. The nuclear enzyme PARP is involved in the DNA damage response [56]. Apoptotic cells show conspicuous changes like nuclear condensation, cell shrinkage, membrane blebbing, and DNA fragmentation. In our study, treatment with THSG suppressed the disruption of the mitochondrial membrane potential, restored cytochrome *c* release, and downregulated the mitochondrial-dependent apoptotic pathway in gentamicin-treated UB/OC-2 cells (Figure 3 and Figure 4). Moreover, we observed that THSG decreased the level of apoptotic cells and cytotoxicity in gentamicin-induced cell damage (Figure 5). These results suggest that THSG might protect mouse cochlear UB/OC-2 cells from gentamicin-induced cell death by inhibiting ROS generation and downregulating the mitochondrial-dependent apoptosis pathway (Figure 6), but further animal experiments are necessary to confirm the otoprotecitive effects of THSG.

## 4. Materials and Methods

### 4.1. Cells and Materials 

The UB/OC-2 cells were purchased from Ximbio (London, UK). Gentamicin was obtained from Standard Chem & Pharm Co., Ltd (Tainan, Taiwan). THSG was purchased from MedChemExpress Company (Monmouth Junction, NJ, USA). MTT was obtained from VWR International (Radnor, PA, USA). LDH cytotoxicity assay kit, DCFDA, Hoechst 33258 dye, JC-1 dye, Annexin V, and PI were purchased from Enzo Life Sciences (Farmingdale, NY, USA). Primary antibodies targeting PARP, cytochrome *c*, β-actin, COX IV, cleaved caspase 9, cleaved caspase 3 were purchased from Cell Signaling Technology (Beverly, MA, USA). HRP-conjugated secondary antibodies were obtained from PerkinElmer Life Sciences (Boston, MA, USA). SOD activity assay kit and the cell fractionation kit were purchased from Abcam (Cambridge, MA, USA).

### 4.2. Cell Culture

The UB/OC-2 cells were grown in MEM GlutaMAXTM medium (Gibco, NY, USA) supplemented with 10% fetal bovine serum (Hyclone Laboratories Inc., Logan, UT, USA) and 50 units/mL interferon-γ (R&D system, Minneapolis, MN, USA). The cells were incubated in a humidified atmosphere with 5% CO_2_ and 95% air at 33 °C.

### 4.3. Cell Viability Assay

The UB/OC-2 cells in 24-well plates (2 × 10^4^ cells/well) were treated with various concentrations of gentamicin (125–1000 μM) for 48 h, and then MTT solution was added to each well at a final concentration of 0.16 mg/mL for 4 h at 33 °C. The formazan crystals were dissolved in dimethyl sulfoxide (DMSO; Sigma-Aldrich, St. Louis, MO, USA) and, finally, a microplate reader (Infinite 200 PRO Series Multimode Reader; TECAN, Zürich, Switzerland) was used to measure the absorbance of each well at 570 nm.

### 4.4. LDH Cytotoxicity Assay

The UB/OC-2 cells in 96-well plates (5 × 10^3^ cells/well) were treated with gentamicin alone or gentamicin with THSG for 48 h. LDH release was performed according to the manufacturer’s instructions for the LDH cytotoxicity assay kit. The absorbance of each well was measured at 490 nm using an Infinite 200 PRO Series Multimode Reader.

### 4.5. Western Blotting Analysis

The UB/OC-2 cells in a 6 cm dish (6 × 10^5^ cells/well) were treated with various concentrations of THSG (5, 10, and 20 μM) for 6 h, followed by 750 μM gentamicin treatment for 48 h. After treatment, UB/OC-2 cells were washed with ice-cold phosphate-buffered saline (PBS) twice, and the total proteins were extracted by lysis buffer (Thermo Fisher Scientific, Waltham, MA, USA). The proteins were resolved using 10–15% sodium dodecyl sulfate-polyacrylamide gel electrophoresis and transferred onto polyvinylidene fluoride (PVDF) membranes (Millipore, Burlington, MA, USA). The membranes were blocked with 3% (*w*/*v*) bovine serum albumin (BSA; Sigma-Aldrich, St. Louis, MO, USA) in Tris-buffered saline with Tween 20 (TBST; VWR International, Radnor, PA, USA) for 1 h at 37 °C and then incubated in related primary antibodies in TBST with 3% (*w*/*v*) BSA overnight at 4 °C. The membranes were incubated with HRP-conjugated secondary antibodies for 1 h at room temperature. Subsequently, protein signal was developed by using enhanced chemiluminescence (Bio-Rad Laboratories, Inc., Hercules, CA, USA) with a KETA C Chemi imaging system (Wealtec Corporation, Sparks, NV, USA).

### 4.6. ROS Detection Assay

The UB/OC-2 cells in 6-well plates (4 × 10^5^ cells/well) were treated with various concentrations of THSG (5, 10, 20 μM) for 6 h, followed by 750 μM gentamicin for 2 h. The cells were incubated with 10 μM DCFDA for 30 min at 33 °C, and then they were collected in PBS. The fluorescence intensity was analyzed using the BD Accuri™ C6 flow cytometry system (BD Biosciences, San Jose, CA, USA).

### 4.7. Determination of SOD Activity

The UB/OC-2 cells were treated with various concentrations of THSG (5, 10, 20 μM) for 6 h, followed by 750 μM gentamicin for 48 h. After the treatments, the cells were lysed, and the activity of the antioxidant enzyme, SOD, was detected using a SOD activity colorimetric assay kit according to the manufacturer’s protocol. The cell pellet was resuspended in 0.1 M Tris/HCl (pH 7.4) containing 0.5% Triton X-100, 5 mM β-ME, 0.1 mg/mL PMSF. The sample extract (20 μL) was added to 20 μL enzyme working solution and 200 μL WST working solution and incubated at 37 °C for 20 min. The absorbance was measured at 532 nm.

### 4.8. Hoechst 33258 Staining

The UB/OC-2 cells in 6-well plates (3 × 10^5^ cells/well) were treated with various concentrations of THSG (5,10, and 20 μM) for 6 h, followed by 750 μM gentamicin for 48 h. Hoechst 33258 dye (20 μg/mL in culture medium) was added to each well for 1 h at 33 °C. Fluorescence images were obtained using an Olympus BX41 microscope (Tokyo, Japan) at the excitation wavelength of 352 nm.

### 4.9. JC-1 Staining

The UB/OC-2 cells in 6-well plates (4 × 10^5^ cells/well) were treated with various concentrations of THSG (5, 10, and 20 μM) for 6 h and subsequently with 750 μM gentamicin for 24 h. The cells were incubated for 10 min at 33 °C with JC-1 dye (5 μg/mL in culture medium). Fluorescence images were obtained using an Olympus BX41 microscope at the excitation wavelength of 515 nm.

### 4.10. Cell Fractionation

The UB/OC-2 cells in 10 cm culture dishes (2 × 10^6^ cells) were treated with various concentrations of THSG (5, 10, and 20 μM) for 6 h and subsequently with 750 μM gentamicin for 48 h. Cytosolic and mitochondrial fractions were executed according to the manufacturer’s instruction. The cells were harvested and resuspended in a buffer containing 0.015% ethylenediaminetetraacetic acid (EDTA) and 0.36% Tris to 2 × 10^7^ cells/mL. The same amount of buffer containing 0.015% EDTA, 0.36% Tris, and 0.001% digitonin was added to the reaction, and then the mixture was incubated for 7 min at room temperature. The samples were centrifuged at 10,000× *g* for 1 min, and the supernatant (cytosolic extract) was transferred to new tubes. The pellets were resuspended in a buffer containing 0.03% EDTA and 0.75% Tris and incubated for 10 min at room temperature. The samples were centrifuged at 10,000× *g* for 1 min, and the supernatant (mitochondrial extract) was transferred to new tubes. Finally, the extracts were stored at −80 °C until use.

### 4.11. Annexin V and PI Double Staining Assay

The UB/OC-2 cells in 6-well plates (4 × 10^5^ cells/well) were treated with various concentrations of THSG (5, 10, and 20 μM) for 6 h and subsequently with 750 μM gentamicin for 24 h. After collection, the cells were incubated with saturating concentrations of Annexin V-FITC and PI (2 μg/mL) in binding buffer for 15 min at room temperature. The results were analyzed by the BD Accuri™ C6 flow cytometry system.

### 4.12. Statistical Analysis

Data were expressed as the mean ± standard deviation of at least three independent experiments. The statistical differences among the groups were analyzed by Student’s *t*-test. A *p*-value of less than 0.05 was considered to be statically significant.

## 5. Conclusions

In summary, this study, to the best of our knowledge, is the first to demonstrate that THSG provides significant protection from gentamicin-induced ototoxicity. Treatment with THSG counteracted gentamicin-induced cytotoxicity and apoptosis through reduction of ROS production, stabilization of mitochondrial membrane potential, downregulation of mitochondrial-dependent apoptosis-related proteins, and upregulation of SOD activity. These findings suggest that THSG could be a potential therapeutic option for the prevention of ototoxicity after gentamicin exposure or as a potential food supplement with antioxidant effects to reduce the prevalence of hearing loss.

## References

[1] WHO Deafness and Hearing Loss. https://www.who.int/health-topics/hearing-loss.

[2] Alvarado J.C., Fuentes-Santamaria V., Melgar-Rojas P., Valero M.L., Gabaldon-Ull M.C., Miller J.M., Juiz J.M. (2015). Synergistic effects of free radical scavengers and cochlear vasodilators: A new otoprotective strategy for age-related hearing loss. Front. Aging Neurosci..

[3] Arslan E., Orzan E., Santarelli R. (1999). Global problem of drug-induced hearing loss. Ann. N. Y. Acad. Sci..

[4] Rybak L.P., Ramkumar V. (2007). Ototoxicity. Kidney Int..

[5] Gonzalez-Gonzalez S. (2017). The role of mitochondrial oxidative stress in hearing loss. Neurol. Disord. Ther..

[6] Fujimoto C., Yamasoba T. (2014). Oxidative stresses and mitochondrial dysfunction in age-related hearing loss. Oxid. Med. Cell Longev..

[7] Ghashghaei S., Emtiazi G. (2013). Increasing the antibacterial activity of gentamicin in combination with extracted polyphosphate from Bacillus megaterium. J. Appl. Microbiol..

[8] Kros C.J., Steyger P.S. (2019). Aminoglycoside- and Cisplatin-Induced Ototoxicity: Mechanisms and Otoprotective Strategies. Cold Spring Harb. Perspect Med..

[9] Quan Y., Xia L., Shao J., Yin S., Cheng C.Y., Xia W., Gao W.Q. (2015). Adjudin protects rodent cochlear hair cells against gentamicin ototoxicity via the SIRT3-ROS pathway. Sci. Rep..

[10] Jiang P., Ray A., Rybak L.P., Brenner M.J. (2016). Role of STAT1 and Oxidative Stress in Gentamicin-Induced Hair Cell Death in Organ of Corti. Otol. Neurotol..

[11] Kalinec G.M., Fernandez-Zapico M.E., Urrutia R., Esteban-Cruciani N., Chen S., Kalinec F. (2005). Pivotal role of Harakiri in the induction and prevention of gentamicin-induced hearing loss. Proc. Natl. Acad. Sci. USA.

[12] Ojano-Dirain C.P., Antonelli P.J. (2012). Prevention of gentamicin-induced apoptosis with the mitochondria-targeted antioxidant mitoquinone. Laryngoscope.

[13] Somdas M.A., Korkmaz F., Gurgen S.G., Sagit M., Akcadag A. (2015). N-acetylcysteine Prevents Gentamicin Ototoxicity in a Rat Model. J. Int. Adv. Otol..

[14] Setz C., Benischke A.S., Pinho Ferreira Bento A.C., Brand Y., Levano S., Paech F., Leitmeyer K., Bodmer D. (2018). Induction of mitophagy in the HEI-OC1 auditory cell line and activation of the Atg12/LC3 pathway in the organ of Corti. Hear. Res..

[15] Kim Y.J., Tian C., Kim J., Shin B., Choo O.S., Kim Y.S., Choung Y.H. (2017). Autophagic flux, a possible mechanism for delayed gentamicin-induced ototoxicity. Sci. Rep..

[16] Kucharava K., Sekulic-Jablanovic M., Horvath L., Bodmer D., Petkovic V. (2019). Pasireotide protects mammalian cochlear hair cells from gentamicin ototoxicity by activating the PI3K-Akt pathway. Cell Death Dis..

[17] Lai R., Li W., Hu P., Xie D., Wen J. (2018). Role of Hsp90/Akt pathway in the pathogenesis of gentamicin-induced hearing loss. Int. J. Clin. Exp. Pathol..

[18] Heinrich U.R., Strieth S., Schmidtmann I., Li H., Helling K. (2015). Gentamicin alters Akt-expression and its activation in the guinea pig cochlea. Neuroscience.

[19] Eshraghi A.A., Van de Water T.R. (2006). Cochlear implantation trauma and noise-induced hearing loss: Apoptosis and therapeutic strategies. Anat. Rec. A Discov. Mol. Cell Evol. Biol..

[20] Kamogashira T., Fujimoto C., Yamasoba T. (2015). Reactive oxygen species, apoptosis, and mitochondrial dysfunction in hearing loss. Biomed. Res. Int..

[21] Noack V., Pak K., Jalota R., Kurabi A., Ryan A.F. (2017). An Antioxidant Screen Identifies Candidates for Protection of Cochlear Hair Cells from Gentamicin Toxicity. Front. Cell Neurosci..

[22] Ling S., Xu J.W. (2016). Biological Activities of 2,3,5,4′-Tetrahydroxystilbene-2-*O*-beta-d-Glucoside in Antiaging and Antiaging-Related Disease Treatments. Oxid. Med. Cell Longev..

[23] Lv G., Lou Z., Chen S., Gu H., Shan L. (2011). Pharmacokinetics and tissue distribution of 2,3,5,4′-tetrahydroxystilbene-2-*O*-beta-d-glucoside from traditional Chinese medicine Polygonum multiflorum following oral administration to rats. J. Ethnopharmacol..

[24] Lv L., Shao X., Wang L., Huang D., Ho C.T., Sang S. (2010). Stilbene glucoside from Polygonum multiflorum Thunb.: A novel natural inhibitor of advanced glycation end product formation by trapping of methylglyoxal. J. Agric. Food Chem..

[25] Bai H.B., Wang J.F., Long J. (2004). Study on optimizing extraction process of root of Polygonum multiflorum. Zhongguo Zhong Yao Za Zhi.

[26] Zhang W., Wang C.H., Li F., Zhu W.Z. (2008). 2,3,4′,5-Tetrahydroxystilbene-2-*O*-beta-d-glucoside suppresses matrix metalloproteinase expression and inflammation in atherosclerotic rats. Clin. Exp. Pharm. Physiol..

[27] Huang C., Wang Y., Wang J., Yao W., Chen X., Zhang W. (2013). TSG (2,3,4′,5-tetrahydroxystilbene 2-*O*-beta-d-glucoside) suppresses induction of pro-inflammatory factors by attenuating the binding activity of nuclear factor-kappaB in microglia. J. Neuroinflam..

[28] Sun T., Liu H., Cheng Y., Yan L., Krittanawong C., Li S., Qian W., Su W., Chen X., Hou X. (2019). 2,3,5,4′-Tetrahydroxystilbene-2-*O*-beta-d-glucoside eliminates ischemia/reperfusion injury-induced H9c2 cardiomyocytes apoptosis involving in Bcl-2, Bax, caspase-3, and Akt activation. J. Cell Biochem..

[29] Zhao J., Xu S., Song F., Nian L., Zhou X., Wang S. (2014). 2,3,5,4′-tetrahydroxystilbene-2-*O*-beta-d-glucoside protects human umbilical vein endothelial cells against lysophosphatidylcholine-induced apoptosis by upregulating superoxide dismutase and glutathione peroxidase. Iubmb. Life.

[30] Han X., Ling S., Gan W., Sun L., Duan J., Xu J.W. (2012). 2,3,5,4′-tetrahydroxystilbene-2-*O*-beta-d-glucoside ameliorates vascular senescence and improves blood flow involving a mechanism of p53 deacetylation. Atherosclerosis.

[31] Wu T.Y., Lin J.N., Luo Z.Y., Hsu C.J., Wang J.S., Wu H.P. (2020). 2,3,4′,5-Tetrahydroxystilbene-2-*O*-beta-d-Glucoside (THSG) Activates the Nrf2 Antioxidant Pathway and Attenuates Oxidative Stress-Induced Cell Death in Mouse Cochlear UB/OC-2 Cells. Biomolecules.

[32] Rivolta M.N., Grix N., Lawlor P., Ashmore J.F., Jagger D.J., Holley M.C. (1998). Auditory hair cell precursors immortalized from the mammalian inner ear. Proc. Biol. Sci..

[33] Rivolta M.N., Holley M.C. (2002). Cell lines in inner ear research. J. Neurobiol..

[34] Weiss S., Gottfried I., Mayrose I., Khare S.L., Xiang M., Dawson S.J., Avraham K.B. (2003). The DFNA15 deafness mutation affects POU4F3 protein stability, localization, and transcriptional activity. Mol. Cell Biol..

[35] Brunetta I., Casalotti S.O., Hart I.R., Forge A., Reynolds L.E. (2012). beta3-integrin is required for differentiation in OC-2 cells derived from mammalian embryonic inner ear. BMC Cell Biol..

[36] Goncalves A.C., Towers E.R., Haq N., Porco J.A., Pelletier J., Dawson S.J., Gale J.E. (2019). Drug-induced Stress Granule Formation Protects Sensory Hair Cells in Mouse Cochlear Explants During Ototoxicity. Sci. Rep..

[37] Xu M.L., Zheng M.S., Lee Y.K., Moon D.C., Lee C.S., Woo M.H., Jeong B.S., Lee E.S., Jahng Y., Chang H.W. (2006). A new stilbene glucoside from the roots of Polygonum multiflorum Thunb. Arch. Pharm. Res..

[38] Han M.N., Lu J.M., Zhang G.Y., Yu J., Zhao R.H. (2015). Mechanistic Studies on the Use of Polygonum multiflorum for the Treatment of Hair Graying. Biomed. Res. Int..

[39] Hata K., Kozawa M., Baba K. (1975). A new stilbene glucoside from Chinese crude drug "Heshouwu," the roots of Polygonum multiflorum Thunb. Yakugaku Zasshi.

[40] Frombaum M., Le Clanche S., Bonnefont-Rousselot D., Borderie D. (2012). Antioxidant effects of resveratrol and other stilbene derivatives on oxidative stress and *NO bioavailability: Potential benefits to cardiovascular diseases. Biochimie.

[41] Lin E.Y., Bayarsengee U., Wang C.C., Chiang Y.H., Cheng C.W. (2018). The natural compound 2,3,5,4′-tetrahydroxystilbene-2-*O*-beta-d*-*glucoside protects against adriamycin-induced nephropathy through activating the Nrf2-Keap1 antioxidant pathway. Environ. Toxicol..

[42] Cheng J., Wang H., Zhang Z., Liang K. (2019). Stilbene glycoside protects osteoblasts against oxidative damage via Nrf2/HO-1 and NF-kappaB signaling pathways. Arch. Med. Sci..

[43] Lee S.Y., Ahn S.M., Wang Z., Choi Y.W., Shin H.K., Choi B.T. (2017). Neuroprotective effects of 2,3,5,4′-tetrahydoxystilbene-2-*O*-beta-d-glucoside from Polygonum multiflorum against glutamate-induced oxidative toxicity in HT22 cells. J. Ethnopharmacol..

[44] Clerici W.J., Hensley K., DiMartino D.L., Butterfield D.A. (1996). Direct detection of ototoxicant-induced reactive oxygen species generation in cochlear explants. Hear. Res..

[45] Abi-Hachem R.N., Zine A., Van De Water T.R. (2010). The injured cochlea as a target for inflammatory processes, initiation of cell death pathways and application of related otoprotectives strategies. Recent Pat. CNS Drug Discov..

[46] El Mouedden M., Laurent G., Mingeot-Leclercq M.P., Taper H.S., Cumps J., Tulkens P.M. (2000). Apoptosis in renal proximal tubules of rats treated with low doses of aminoglycosides. Antimicrob. Agents Chemother..

[47] Jia Z., He Q., Shan C., Li F. (2018). Tauroursodeoxycholic acid attenuates gentamicin-induced cochlear hair cell death in vitro. Toxicol. Lett..

[48] Zhou M., Sun G., Zhang L., Zhang G., Yang Q., Yin H., Li H., Liu W., Bai X., Li J. (2018). STK33 alleviates gentamicin-induced ototoxicity in cochlear hair cells and House Ear Institute-Organ of Corti 1 cells. J. Cell Mol. Med..

[49] Wang J., Ladrech S., Pujol R., Brabet P., Van De Water T.R., Puel J.L. (2004). Caspase inhibitors, but not c-Jun NH2-terminal kinase inhibitor treatment, prevent cisplatin-induced hearing loss. Cancer Res..

[50] Li P., Nijhawan D., Budihardjo I., Srinivasula S.M., Ahmad M., Alnemri E.S., Wang X. (1997). Cytochrome *c* and dATP-dependent formation of Apaf-1/caspase-9 complex initiates an apoptotic protease cascade. Cell.

[51] Guo C., Sun L., Chen X., Zhang D. (2013). Oxidative stress, mitochondrial damage and neurodegenerative diseases. Neural Regen. Res..

[52] Denamur S., Tyteca D., Marchand-Brynaert J., Van Bambeke F., Tulkens P.M., Courtoy P.J., Mingeot-Leclercq M.P. (2011). Role of oxidative stress in lysosomal membrane permeabilization and apoptosis induced by gentamicin, an aminoglycoside antibiotic. Free Radic. Biol. Med..

[53] Bodmer D., Brors D., Pak K., Bodmer M., Ryan A.F. (2003). Gentamicin-induced hair cell death is not dependent on the apoptosis receptor Fas. Laryngoscope.

[54] Guan M.X. (2004). Molecular pathogenetic mechanism of maternally inherited deafness. Mitochondrial Pathogenesis.

[55] Lin C.D., Kao M.C., Tsai M.H., Lai C.H., Wei I.H., Tsai M.H., Tang C.H., Lin C.W., Hsu C.J., Lin C.Y. (2011). Transient ischemia/hypoxia enhances gentamicin ototoxicity via caspase-dependent cell death pathway. Lab. Investig..

[56] Joza N., Susin S.A., Daugas E., Stanford W.L., Cho S.K., Li C.Y., Sasaki T., Elia A.J., Cheng H.Y., Ravagnan L. (2001). Essential role of the mitochondrial apoptosis-inducing factor in programmed cell death. Nature.

